# Under the (legal) radar screen: global health initiatives and international human rights obligations

**DOI:** 10.1186/1472-698X-12-31

**Published:** 2012-11-15

**Authors:** Rachel Hammonds, Gorik Ooms, Wouter Vandenhole

**Affiliations:** 1 Institute of Tropical Medicine, 155 Nationalestraat, 2000, Antwerp, Belgium; 2 Faculty of Law, University of Antwerp, Stadscampus, S.V.132 Venusstraat 23, 2000, Antwerp, Belgium

**Keywords:** Global health initiatives, Human rights, The Global Fund to fight AIDS, Tuberculosis and Malaria, HIV, Right to health, Transnational legal process, Extraterritorial legal obligations

## Abstract

**Background:**

Given that many low income countries are heavily reliant on external assistance to fund their health sectors the acceptance of obligations of international assistance and cooperation with regard to the right to health (global health obligations) is insufficiently understood and studied by international health and human rights scholars. Over the past decade Global Health Initiatives, like the Global Fund to fight AIDS, Tuberculosis and Malaria (Global Fund) have adopted novel approaches to engaging with stakeholders in high and low income countries. This article explores how this experience impacted on acceptance of the international obligation to (help) fulfil the right to health beyond borders.

**Methods:**

The authors conducted an extensive review of international human rights law literature, transnational legal process literature, global public health literature and grey literature pertaining to Global Health Initiatives. To complement this desk work and deepen their understanding of how and why different legal norms evolve the authors conducted 19 in-depth key informant interviews with actors engaged with three stakeholders; the European Union, the United States and Belgium. The authors then analysed the interviews through a transnational legal process lens.

**Results:**

Through according value to the process of examining how and why different legal norms evolve transnational legal process offers us a tool for engaging with the dynamism of developments in global health suggesting that operationalising global health obligations could advance the right to health for all.

**Conclusions:**

In many low-income countries the health sector is heavily dependent on external assistance to fulfil the right to health of people thus it is vital that policies and tools for delivering reliable, long-term assistance are developed so that the right to health for all becomes more than a dream. Our research suggests that the Global Fund experience offers lessons to build on.

## Background

“If we are to understand the significance of international law and how it works and evolves, it is essential to look outside of the law itself.” Oscar Schachter [1]

The first decade of the 21^st^ century brought substantial change to the global health aid architecture; including an increase in the number of actors and, most notably, the emergence of well-funded dynamic global health initiatives (GHIs), like the Global Fund to Fight AIDS, Tuberculosis and Malaria (the Global Fund) and the GAVI Alliance, focused mainly on communicable diseases and vaccines. Yet, as the world enters the second decade of the new millennium the maternal mortality rate in Afghanistan is 400 times that of Italy, stark evidence of the consequences of the ongoing disparity between respect and fulfilment of basic human rights in high and low income countries ^1^. The legal scholar Lawrence Gostin has characterized these massive health disparities as an “unconscionable gap” noting that “the avoidable suffering and early death among the world’s least healthy people… is a breach of social justice that is no longer ethically acceptable” [2]. The parameters of the obligation of international assistance and cooperation, as defined in the International Covenant on Economic, Social and Cultural Rights (ICESCR), [3] in addressing this disparity remain hotly debated ^2^.

The past decade has also witnessed legal scholars beginning to address the challenges raised by the human rights obligations of states beyond their borders, which are referred to as extraterritorial or transnational obligations ^3^. With the aim of clarifying the scope of national and international obligations under the ICESCR the United Nations Committee on Economic, Social and Cultural Rights (the Committee) has issued various general comments, including one on the right to health [4]. General Comment 14 on the right to health and the work of the United Nations Special Rapporteurs on the right to health have contributed to improving the understanding of the importance and scope of obligations of international assistance and cooperation with regard to the right to health, which we shall refer to as global health obligations ^4 and 5^.

The global health policy scholar Benjamin Mason Meier argues that as the global health landscape continues to evolve the increasing influence of norms, including human rights law, on global health policy and institutions is evident [5]. This article explores both how and why global health obligations founded on human rights principles can be operationalised thus advancing the right to health. To examine how this might occur in practice we present the results of our study examining the impact of one key GHI, the Global Fund, on how different actors characterized their global health obligations on a continuum from charitable to legal obligation. Our work was guided by our research question, “is the evolution of the Global Fund evidence of a growing acceptance of the international obligation to (help) fulfil the right to health beyond borders?”

During the past decades, the study of compliance has assumed a prominent role in international legal studies ^6^. Such work is clearly useful for enhancing scholars’ understanding of international law and its potential to change state behaviour. However, we agree with Teitel and Howse that a compliance centric approach is too narrow leading to inadequate scrutiny and “a tendency to oversimplify if not distort the relation of international law to politics” [6]. It can be argued that for most legal scholars and states “the rules” are not yet clear as regards the nature and scope of global health obligations ^7^. Thus our analysis moves beyond the strict compliance based framework instead focusing on how and why states move towards an understanding of what compliance with international law entails. As such our analysis focuses on how and why the international legal, political and social environments in which states and non-state actors interact can impact on the movement towards respect for global health obligations. In particular we shall argue that the traditional international law approach; focused on general principles of international law, the role of treaties and customary international law (the sources of international law) ^8^, fails to capture key elements related to the complexity and consequences of this interactive process.

We also employed a non-traditional legal research methodology, exploring the engagement of the Global Fund with different actors and stakeholders in two high income states and one multilateral institution. Through a series of semi-structured stakeholder interviews, we explored if and why interaction with the Global Fund has changed the way in which high income states characterize their global health obligations on a spectrum from charity to legal obligation. We argue that focusing solely on compliance related behaviour and speech, would fail to capture the normative effect of international human rights law on current practice, as it moves along the path towards or away from acceptance of global health obligations. To help capture the significance of this complexity, our work engages with a concept that first evolved in non-legal theory -social constructivism -to help explain how significant legal change may arise through a process of normative internalization; specifically the acceptance of global health obligations by states. We argue that Howard Honghu Koh’s approach to transnational legal process and his tripartite framework of ‘interaction, interpretation and internalization’ help provide insights into what Berman terms “the long process of rhetorical persuasion” [7,8].

As the global health and political landscapes are constantly changing there are limitations to our “snapshot in time” approach ^9^. However, we believe that this research provides interesting insights into how and why different policy stakeholders (including policy makers and implementers, lobbyists and politicians) engage with the concept of global health obligations. We shall argue that the interaction high income states engage in with respect to the Global Fund suggests the normative and law generating potential of such behaviour and as such this type of analysis is useful for those interested in examining that vast terrain between international assistance and cooperation as a form of charity and the acceptance of it as a legal obligation.

## Methods

To improve our understanding of the impact of the Global Fund on stakeholder thinking and behaviour; in particular that of high income states, we embarked on a qualitative study within a transnational legal process paradigm ^10^. An extensive document review of relevant legal, public health and grey literature informed our questions and analysis [9]. Following our literature review we developed interview questions to assist us in further understanding stakeholder thinking and behaviour. We then identified key informants from health and development stakeholders engaged with one of three high income actors; Belgium, the executive arm of the European Union (EU), the European Commission and the United States. We selected the European Commission, and the United States because they are two of the more influential actors in so far as they rank among the Global Fund’s biggest funders and have a representative on the Global Fund Board. We selected Belgium because it is a State Party to the ICESCR and we were interested in comparing the behaviour and motivations of a Party to the ICESCR with the United States, which is not a State Party ^11^. A second goal of these interviews was to introduce a “reality check” into our research question “is the evolution of the Global Fund evidence of a growing acceptance of the international obligations to (help) fulfil the right to health beyond borders?”

For all three actors the key informants included civil society representatives, key figures in their respective executive bodies and key individuals involved in the administration and implementation of policy. For one of the actors we interviewed an elected representative.

We conducted 19 semi-structured (eight classified as American, seven classified as European and four classified as Belgian) interviews with open-ended questions. We used the following quote from the former UN Special Rapporteur on the Right to Health, [4] Paul Hunt to frame the questions, “if there is no legal obligation underpinning the human rights responsibility of international assistance and cooperation, inescapably all international assistance and cooperation is based fundamentally upon charity. While such a position might have been tenable 100 years ago, it is unacceptable in the twenty-first century” [10]. All of the interviews were recorded, anonymised and transcribed verbatim. Data analysis involved three stages, first order descriptive analysis which involved repeated reading of the transcripts to develop familiarity with the content of the interviews. The second-stage involved thematic analysis in which responses, where relevant, were grouped according to the core obligations framework outlined below. Each of these themes flows from the core obligations relating to health namely [4];

1. ‘It is particularly incumbent’ → it is a legal obligation and not a matter of charity or political choice

2. ‘States parties and other actors in a position to assist’ → shared responsibility which implies a form of burden sharing is needed

3. ‘Enabling developing countries’ → focused on countries that lack domestic capacity

4. ‘Fulfil core and other obligations’ → not limited to AIDS, TB and malaria

### Theoretical analysis

We then engaged in the third order theoretical analysis of the interviews, in which we apply the transnational legal process framework of interaction, interpretation and internalization to assess the second order thematic analysis for evidence of patterns, ideas that stood out and those responses that were novel. It is worth highlighting that this is not a strictly linear process. A specific interaction may lead to a specific interpretation but these steps do not necessarily lead to internalization. Thus, as will be described in detail below, in analysing our interviews we looked for references to repeated instances of interaction and interpretation that could eventually generate a norm that may in turn become internalized ^12^.

## Results

This section of the article explores the key concepts we extracted from our extensive literature review. The first subsection examines the legal obligations of international cooperation and assistance with respect to the right to health (global health obligations) focusing on the concept of core obligations. The second sub-section explores the political and social context in which the GHIs came into existence, with a particular focus on the background, structure and functioning of the Global Fund. In the third subsection we introduce Koh’s theory of transnational legal process.

### The right to health

To examine the legal obligations of international cooperation and assistance with respect to the right to health (global health obligations) we start with an overview of the fundamental international law concepts key to our analysis. Second, we review the legal basis of the right to health in international law. Next, we address the core content of the right to health as articulated in General Comment 14 highlighting several key features that we focused on in our interviews and subsequent analysis. We shall explore how this core content may be viewed as a partial clarification of global health obligations, helping us to better understand the universal minimum legal claim that all people have.

#### International law

Traditionally the sources of international law have been understood to include international treaties and conventions, customary practice of states and general principles of law ^8^. It is generally accepted that international law can be created through treaties (binding legal agreements between states) and customary law (rules that evolve over time, becoming commonly accepted through continuous practice) [1]. This approach implies that binding legal obligations can only flow from such sources both of which require state consent. Such a conservative interpretation has guided much, but not all, international legal scholarship [11]. Some suggest that a more progressive approach flows from general principles of international law arguing they have the potential to embrace actors other than states (e.g. NGOs and corporations). Proponents contend that if there is a general expectation in society (e.g. human rights norms) this can become a principle of international law that can eventually bind a range of actors ^13^.

In the human rights field the impact of globalization has led some to question the conservative, state-centric framework of international law. The legal scholar Oscar Schachter noted that international law “is more than a given body of rules and obligations. It involves purposive activities undertaken by governments directed to a variety of social ends.” [1]. Some scholars have moved beyond the state-centric framework to address the obligations of non-state actors including non-governmental organizations and corporations, [12-15]. Others, like Berman and Koh have looked to a more expansive understanding of how law develops. Our analysis moves beyond the strict compliance based framework drawing heavily on Koh’s theory of transnational legal process as part of what Berman terms “the long process of rhetorical persuasion” [7,8,11,16].

It is important for our purposes to highlight the fact that a large and influential body of international law is not considered to be legally binding; it is not ‘hard law’. Such law is referred to as ‘soft law’ and includes non-binding declarations (like the Declaration on the Right to Development [17]); recommendations; guidelines and the general comments issued by the United Nations Committee on Economic Social and Cultural Rights, discussed below. Even though soft law instruments are not legally binding, there remains a strong expectation that their provisions will be respected and followed by the international community [18].

The process by which soft law instruments can over time ‘harden’ into binding law is highly complex and controversial. Instead of addressing the intricacies of these arguments in relation to our topic we shall instead adopt a transnational legal process based analysis. In sections three and four we shall examine the potential jurisgenerative power of interaction among multiple international actors to examine how soft law might impact on the international legal consensus, in particular with respect to health.

#### Legal instruments recognizing the right to health

The first international legal document recognizing an individual right to health was the 1946 World Health Organization (WHO) Constitution ^14^. It is important to note that the WHO Constitution fails to clarify what such adequate measures may be. The non-binding 1948 Universal Declaration of Human Rights includes a wide range of civil, political; economic, social and cultural rights including a reference to the right to health ^15^. Subsequently, the right to the highest attainable standard of health has been reaffirmed as a universal human right and is enshrined in five ^16^ of the key international human rights treaties.

The principle legal framework for understanding governmental obligations relating to the right to health is found in the ICESCR [3]. It is worth stressing that under international law the ICESCR is an international treaty and as such is considered binding on States parties. Article 12 of the ICESCR broadly defines the right to health as “the right to the highest attainable standard of physical and mental health.” It provides limited guidance regarding the scope of the right and the obligations of States parties including reducing the stillbirth rate and infant mortality, preventing, treating and controlling epidemic, endemic, occupational and other diseases and creating conditions which would assure to all medical service and medical attention in the event of sickness. This guidance has been refined in later conventions and through Committee jurisprudence including most significantly in General Comment 14, discussed below [4].

#### International cooperation and assistance

The concept of international cooperation in helping to achieve universal respect for human rights has its basis in Articles 55 and 56 of the 1945 Charter of the United Nations (UN Charter) ^17^, a formal and authoritative foundation for future declarations and treaties with respect to international cooperation.

For our purposes, it is important to note that three of the key international human rights treaties refer to the importance of international assistance and cooperation with respect to achieving the rights in the treaties namely the ICESCR (article 2.1) ^18^, the Convention on the Rights of the Child (CRC) ^19^ and the most recent international human rights treaty; the Convention on the Rights of Persons with Disabilities (CRPD) ^20^. However as Bueno de Mesquita, Hunt and Khosla note, “Like many elements of human rights, the parameters of international assistance and cooperation in economic, social and cultural rights are not yet settled” [19].

#### General comment 14 on the right to health

As noted above General Comment 14, acts as an authoritative interpretative guide to the right to health providing much needed guidance as to its normative scope and the obligations of States parties ^4^. The combination of the General Comment, the work of the UN Special Rapporteurs on the right to health and the ongoing work of the Committee, including in its concluding observations to States parties, provide further clarification. In addition to addressing the scope of the right General Comment 14 para. 45 affirms the importance of international cooperation in achieving the right. With respect to international cooperation it pronounces:

For the avoidance of any doubt, the Committee wishes to emphasize that it is particularly incumbent on States parties and other actors in a position to assist, to provide “international assistance and cooperation, especially economic and technical” which enable developing countries to fulfil their core and other obligations.

This refinement of the scope of duties of ‘international assistance and cooperation’ extended our understanding beyond the more general statement in article 2(1) of the ICESCR which failed to distinguish sufficiently between national and international obligations. By clarifying that “core obligations” relating to the right to health must be fulfilled and that international assistance and cooperation may, under certain circumstances, have a complementary or supplementary role to play in their fulfilment the Committee advanced the interpretation that the right to the highest attainable standard of health is a universal right that can empower disadvantaged individuals and communities ^21^. In the words of Hunt and Backman it grants “entitlements which place legal and moral obligations on others.” [20]. However, as we shall discuss below these entitlements are circumscribed not unlimited but the boundaries of these limits require greater precision.

#### Core obligations

The right to health is a social right and as such the obligations it imposes on States parties are to be realized progressively and in accordance with a state’s available resources ^22^. The Committee addressed the contention that “progressive realization” might be used by states to avoid respecting, protecting and fulfilling their obligations and imply that economic and social rights had no “immediate obligations” in General Comment 3, on the Nature of State Parties Obligations [21]. General Comment 3 elucidates a series of concepts and principles that define the nature of states’ obligations, including the principle of non-discrimination, the principle of non-retrogression (a state must not take steps backwards), and the concept of core obligations ^23^.

The concept of core obligations is fundamental to our analysis as it straddles the boundary between the obligations of a State Party and the obligations of those in a position to assist; the wealthy members of the international community. In General Comment 14 the Committee defined the core obligations that arise from the right to health as including obligations to ensure access to essential health facilities and goods and services on a non-discriminatory basis, to develop and implement a national public health plan that addresses the health needs of the entire population through a transparent and participatory process and the promotion of the preconditions of health ^24^. As our later analysis draws on the specific elements of General Comment 14 we wish to highlight that essential health services includes the provision of essential drugs, as defined by the WHO. The WHO list of essential drugs includes anti-retroviral (ARV) treatment for people living with HIV.

Paragraph 47 of General Comment 14 clearly states that these core obligations are not subject to the principle of progressive realisation; they are of immediate effect ^25^. In essence they establish a floor or minimum package of health services that all people in the world have the right to immediately and which all states must provide, irrespective of available resources ^26^. This interpretation has implications for both a state that is unable to fulfil its core obligations and those states in a position to assist. The current reality is that most low-income countries are too poor to fulfil the core obligations or even offer a basic package of health services for all, which the WHO estimates would cost US$40 per person per year ^27^. For some states the cost of providing childhood vaccinations would exhaust their annual national health budget. What then is the role of states in a position to assist?

#### The role of states in a position to assist (high income states)

Given the vast global economic disparities the international community must have a role in fulfilling core obligations on a global scale if they are to be a reality for low income, vulnerable and disadvantaged people. In addressing the role of states in a position to assist several key questions must be answered. First, what is the scope of their obligation, i.e. what does this core obligation include, and when is it satisfied? Second, when is this obligation triggered?

As noted above, in General Comment 14 the Committee offers guidance on the scope of the obligation which includes, among other things, developing and implementing a national public health plan addressing the health needs of the entire population through a participatory and transparent process, ensuring access to essential medicines, maternal, child and reproductive health care, immunizations against major infectious diseases and access on a non-discriminatory basis to health facilities, good and services. This list does not suggest a one-size fits all answer to the core content or obligation and as the Joint Action and Learning Initiative makes clear “the essential package of health goods and services will not be the same in all countries, requiring flexibility and state authority (through inclusive processes to define priorities), but still within acceptable standards.” ^5^. Additional work is also required to establish when this obligation is satisfied. As we shall argue below the recent experience with the Global Fund suggests one avenue for answering this question.

General Comment 14 reaffirms that the state is the primary actor responsible for delivering the right to health for those on its territory regardless of the availability of its resources. We argue that the international obligation for states in a position to cooperate and provide assistance is a complementary or supplementary obligation that does not limit or qualify the primary obligation of the state to spend the maximum of available resources towards achieving the right to health, and all other rights, not just the minimum core [19,22]. Thus, in assessing the ability of low-income countries to fulfil their core obligations, one should not only consider a state’s domestic resources but also resources they receive through international assistance and cooperation. This interpretation is affirmed by the comments of former UN Special Rapporteur on the Right to Health Paul Hunt’s who noted that the list of core obligation and obligations relating to international cooperation under article 2.1 of the Covenant “should be seen as two halves of a package” [23].

The supplementary or complementary obligation of the international community is a limited obligation triggered when a state demonstrates that despite its best efforts it is unable (not unwilling) to fulfil its core obligations ^30^. The question of when a state has expended its maximum available resources is complicated, controversial and can be highly political ^31^. Further, where domestic resource constraints may prevent a state from realizing the core content of the right to health it has the obligation to request international assistance and cooperation from those in a position to assist. In determining the form of such assistance and cooperation in General Comment 19 the Committee has argued that the core obligations are to be prioritized [24].

The remainder of this article reflects our attempt to assess how recent experience with new global health actors has impacted on the approach of high income states, specifically whether this has impacted on their interpretation of obligations to cooperate and provide assistance to realize the right to health or even to internalize new norms. We chose to focus on the interaction of global health actors (high income states, global health advocacy groups and academics) with a fairly new global health player, the Global Fund and we now turn to a brief overview of its set-up and functioning.

### The emergence of the Global Fund

This subsection explores the political and social context in which the GHIs came into existence, with a particular focus on the background, structure and functioning of the Global Fund.

#### Background

As Alan Whiteside notes, AIDS is new; 2011 is the thirtieth anniversary of the first public report on AIDS in the Atlanta (US) based Centers for Disease Control Morbidity and Mortality Weekly Report ^32^[24,25]. The initial panic and fear around AIDS stemmed in large part from the ease of transmission and absence of treatment. An HIV diagnosis was a death sentence. This pessimism changed in 1996 when the discovery of effective anti-retroviral therapies (ART) was announced at the Vancouver International AIDS Conference. For those able to afford treatment, HIV became a serious but often manageable chronic disease. However HIV has not spread uniformly across the globe. It has spread most rapidly through populations in countries which can not afford treatment for their people and through populations whose human rights are often not-respected, including women, men who have sex with men and intravenous drug users ^33^.

In the 1980s and 1990s the push to find a treatment and cure for HIV was spearheaded by activists from the American gay community; the most affected community in the US at that time. This life or death struggle against marginalization, discrimination and stigma resulted in a new form of intense activism that effectively linked health and human rights. Having pushed for pharmaceutical companies to intensify research for a treatment and fought for such treatment to be accessible in their home country (i.e. funded by insurers, easy and reliable access for all) AIDS activists broadened their fight to push for access to treatment for all; American, Ugandan, Vietnamese or South African. They pushed for HIV/AIDS to feature prominently on the global agenda and at a 2000 G8 summit meeting in Okinawa, Japan, G8 leaders acknowledged the need for significantly greater resources to respond to AIDS, tuberculosis and malaria [26]. In 2001 at an African Summit on AIDS then UN Secretary General Kofi Annan called for the creation of a global trust fund to raise additional money. In June 2001 a UN General Assembly Special Session on AIDS committed to creating such a fund [27]. A transitional working group was formed and in January 2002 the permanent secretariat of the Global Fund to Fight AIDS, Tuberculosis and Malaria (Global Fund) was established in Geneva, Switzerland. Three months later the Global Fund Board approved the first round of grants to 36 countries.

#### The Global Fund

For our purposes several key features of the Global Fund are of interest namely its role as a financial enabler, not as an implementer, the fact it is a global public/private partnership and its emphasis on transparency.

As noted above, the Global Fund was created in 2002 “to dramatically increase resources to fight three of the world's most devastating diseases, and to direct those resources to areas of greatest need.” ^34^. It is an international financing mechanism that funds programs through grants not loans; to address three diseases, HIV, tuberculosis and malaria. As such it finances vertical interventions that target three diseases not health systems as a whole. It is not a norm setting body; this is generally the role of the WHO.

Like the Global Alliance for Vaccines and Immunisations (GAVI) Alliance; the Global Fund approach is innovative in that it is a partnership among governments, the private sector (including pharmaceutical companies and foundations like the Gates Foundation), civil society and affected communities [28]. This approach is reflected at both the global and country level.

The Global Fund is committed to the transparency and accountability of its operations; including those of grant recipients and grant contributors. It is a result-driven organization and provides easy access (i.e. it is available online) to its funding data. It also publishes details about the pledges and financing it receives; as well as details about its grants and implementation of these grants. This transparency is vital as it allows civil society activists to monitor what their governments commit to and hold them to account.

The Global Fund does not link a particular donor to a particular project or country. Instead it pools funds from high income countries funds and funds proposals based on merit not political considerations [29]. In theory the modus operandi of the Global Fund should help to enhance country ownership of projects. Instead of drafting project proposals for countries the Global Fund Board issues calls for proposals; countries apply for funds through a national-level Country Coordinating Mechanism (CCM) which includes representatives from governments, multilateral and bilateral agencies, non-governmental organizations (NGOs), people living with the diseases, academic institutions, and private businesses ^35^. The proposals are reviewed by the independent Technical Review Panel, which makes recommendations to the Global Fund Board. The Global Fund Board includes representatives of both high income countries and recipient governments, NGOs from the South and the North, the private sector and communities affected by the diseases. In theory, this highly inclusive structure should allow for a greater balance between the interests of high income countries and low income countries ^36^.

Once the Global Fund Board approves a proposal, a grant agreement is signed with a Principal Recipient, proposed by the CCM. A Local Fund Agent oversees implementation, acts as an independent auditor of expenditure and activities, and liaises with the Global Fund Secretariat. The Global Fund Secretariat oversees the day to day operations among other things managing the proposal applications process, raising money from the public and private sectors and reporting to the Board and the public on Global Fund activities. Attempts to formalize high income countries burden sharing have not yet been successful ^37^.

The Global Fund’s mandate does not refer to the right to health. It was established to act as an international financing mechanism to help finance the fight against three diseases. It is structured to be flexible, efficient, country-driven and results focused. It is important to note that the Global Fund does not claim to play a role in fulfilling the right to health. References to human rights in its documents tend to be limited to discussion of principles of non-discrimination. The analysis we shall develop in the sections 3 and 4 shall attempt to show that although the Global Fund is silent about its role as a financing instrument that allows countries to fulfil the right to health it has to a certain extent played such a role.

### Moving beyond compliance-transnational legal process

Schachter asserted that “Much compliance can be attributed to institutionalized habit; officials follow the rules as a matter of practice,” [1] As we have noted above it can be argued that for most legal scholars and states “the rules” are not yet clear as regards the nature and scope of global health obligations. In section three we explore how engagement in the international political process can shape the evolution of the international consensus on particular legal obligations. Our analysis thus moves beyond the strict compliance based framework drawing heavily on Koh’s theory of transnational legal process [16], [7], [8].

#### International law-moving beyond compliance ^38^

Over thirty years ago in his book *How Nations Behave* the American Professor Louis Henkin claimed that “almost all nations observe almost all principles of international law and almost all of their obligations all of the time” [30]. Challenging or supporting his assertion is certainly an interesting worthwhile field of study. However taking our inspiration from Koh we find the richer questions to be-why do nation states and other transnational actors obey international human rights law, and why do they sometimes disobey it [16], [7], [8]? As such we have chosen to move beyond examining compliance alone and instead focus on the process by which legal actors come to internalize and comply with legal norms or participate in the generation of new norms, namely transnational legal process ^39^.

When examining compliance most international legal and international relations scholars have focused their analysis on two distinct mechanisms by which law changes state practice; the realist paradigm which examines power and coercion and that of persuasion ^40^. The persuasion school has developed in several different directions. It includes scholars who focus on the long-term self-interest of states in obeying the law, and those who attempt to capture the powerful influence of ideas, norms and social forces that shape state behaviour, including compliance [31]. Several liberal international relations scholars have contributed Kantian inspired compliance theories tied to the role of a law abiding national identity ^41^.

The constructivists have taken a different approach arguing that norms play a key role in forming national identity and that compliance with international law may form part of a nation’s identity [32,33]. The engagement of international relations with social constructivism began in the 1980s as a reaction to the neorealist focus on the distribution of power and national interest as the drivers of state behaviour and the rejection of the idea that anarchy leads to self-help [34]. Alexander Wendt, a leader in bringing constructivism into international relations stresses that it is not simply about adding the role of ideas to existing theories of international relations. Martha Finnemore’s work extends the focus to the norms of international society and the way in which they shape state interests and identity. She argues that international norms promoted by international organizations can influence national behaviour. Her ground-breaking case-study on the role of the World Bank argues how the acceptance by developing states of the World Bank discourse on poverty alleviation as a norm of economic policy can not be explained solely by a neorealist focus on power [32,33].

Koh claims that these explanations of why nations obey or comply with international law; power, national interest, national identity and identity formation, are both persuasive and complementary but that they fail to fully explain why nations obey international law. He argues that a full answer must address the importance of interaction, interpretation of international norms and domestic internalization of international norms as determinants of compliance [16], [7], [8]. Thus to understand the complexities of why nations comply with international law including, international human rights law, Koh claims we also need to understand the role of transnational legal process ^42^.

#### Transnational legal process

The theory of transnational legal process can be traced to what has been termed the “new” New Haven School of international law that emerged in the 1990s ^43^. Its approach and focus is inspired by the original New Haven School which as Janet Koven Levit summarized; “offered a kind of socio-legal realism to combat the power-based realism that had dominated the early Cold War period” ^44^. Koh and colleagues were also influenced by legal pluralism and the work of Robert Cover, who ironically enough, did not focus on international law. However as a legal pluralist Cover argued that law is constantly constructed through a contest waged among various norm-generating communities [35]. The other major influence was the changes in the international political arena, foremost increased globalization and the rise of myriad transnational actors. As such the theory moves beyond the traditional state-centric model to look at the world as one of multiple normative communities which shape the emergence of legal norms to varying degrees.

As our analysis shall be guided by Koh’s definition of transnational legal process it is useful to reproduce it in full. For Koh:

Transnational legal process describes the theory and practice of how public and private actors-nation-states, international organizations, multinational enterprises, non-governmental organizations and private individuals-interact in a variety of public and private, domestic and international fora to make, interpret, enforce, and ultimately, internalize rules of transnational law [16], [7], [8].

Koh argues that transnational legal process has four distinct features. Firstly, it is non-traditional, as discussed above it moves beyond the traditional dichotomies of public/private and domestic/international. Second, it embraces a non-statist approach, acknowledging that actors involved in the process include both state and non-state actors. Third, it is a dynamic process that is multidirectional in so far as the process of transformation can be from non-state actor to state and back. Finally, it is also normative. The process of interaction generates new norms which are interpreted, enforced and internalized. For our analysis the salient feature of transnational legal process is the fact the theory embraces the normativity of the process ^45^.

Koh stresses that internalization takes place at three levels; social, political and legal. Social internalization refers to a norm that is widely accepted by the public as legitimate and results in general obedience (e.g. global racial equality). Political internalization occurs when a norm is accepted by political elites and forms part of policy (e.g. development assistance). Finally legal internalization occurs when a norm is incorporated into the domestic legal system through legislation, judicial interpretation or executive action. The interaction among these different forms of internalization can be complex ^46^.

## Discussion

### Transnational legal process and the Global Fund

In this section we explore a practical example assessing how ongoing engagement with an international institution like the Global Fund can impact on the international legal consensus. We use Koh’s tripartite framework of interaction, interpretation and internalization to analyze how and why engagement with the Global Fund has, or has not had any impact on the nature of the engagement of high income states with their global health obligations.

### A New forum for stakeholder interaction

Applying the transnational legal process theoretical framework to our research enhances our ability to understand why different actors take the decisions they take with respect to their interaction with the Global Fund. To understand how our case study on the Global Fund, can be understood through the transnational legal process lens consider the following.

By the mid 1990s the response to the global AIDS epidemic was becoming an important political and human rights issue. The creation of a new institution in 2001, the Global Fund, to help disburse funds so as to start down the path towards universal access to anti-retroviral (ARV) treatment was welcomed by AIDS activists. From the transnational legal process perspective the creation of the Global Fund provided a new forum for stakeholder interaction. It is not part of the United Nations system, nor is it an arm of the WHO or World Bank. It provided an opportunity for interaction that would provoke a reaction-an ongoing dialogue and interpretation of what countries in a position to assist should do to respond to HIV, tuberculosis and malaria.

The institutional structure of this new global institution was and is unique (see details in section two). The requirements of different funding rounds evolve according to scientific evidence on best practice and stakeholder demands. The specific forum for interaction and decision-making, the Global Fund Board, has a novel structure in comparison to traditional development actors like the World Bank or bilateral development assistance actors. The inclusion of private sector, state and civil society actors from both high and low income states on the Global Fund Board reflects the growing role of non-state actors on the global stage. Gostin and Sridhar affirm the importance of this broader approach arguing that the WHO, the global health norm setting agency, would be more effective by “giving voice and representation to key stakeholders, including philanthropies, businesses, public/private partnership and civil society” [28].

As such the Global Fund exemplifies each of the features of transnational legal process outlined above. It is a both a non-traditional, non-statist actor in the development field bringing together a wide-range of actors that often sit on opposite sides of the table; for example civil society and the private sector. It is dynamic and rapidly evolving to respond to demands of states as well as non-state actors. Finally, we shall argue that our interviews suggest that this interaction is normative and potentially jurisgenerative. As Koh argues, “Transnational legal process matters because it increasingly influences law and policies that govern us, particularly through processes by which international law and policies become domesticated into US law and polices” ^43^.

### Examining the process-the interviews

This analysis draws from 19 semi-structured interviews, conducted from June 2010 to February 2011, with stakeholders with ties to two high income states and those who represent or lobby key European Union (EU) institutions, and in particular the European Commission. The interviews lead us to insights that move beyond the nature of that process to examine the normativity of that process arguing that the practice of ongoing engagement with the Global Fund suggests the potential “jurisgenerative” or law-creating effect that such a process can have on conduct ^49^.

Our discussion centres on the interviewees’ responses, which were grouped according to the core obligations framework outlined below. Each of these themes flows from the core obligations relating to health namely;

1. ‘It is particularly incumbent’ → it is a legal obligation and not a matter of charity or political choice

2. ‘States parties and other actors in a position to assist’ → shared responsibility which implies a form of burden sharing is needed

3. ‘Enabling developing countries’ → focused on countries that lack domestic capacity

4. ‘Fulfil core and other obligations’ → not limited to AIDS, TB and malaria

We then apply the transnational legal process framework of interaction, interpretation and internalization to the grouped responses looking for evidence of patterns, ideas that stood out and those responses that were novel.

### Interaction and interpretation-the Global Fund and core obligations

Koh suggests that the interaction between states and the transnational legal process can encourage compliance with international human rights obligations [16], [7], [8]. In the following sub-sections the interviews shall serve as the basis for evaluating the impact of the Global Fund on the degree of acceptance of international obligations of assistance and cooperation to help fulfil a limited element of the core content of the right to health. Again, we reiterate that there are limitations to this approach but believe it enhance understanding of how global health obligations can be operationalised.

#### International assistance and cooperation is a legal obligation and not a matter of charity or political choice

The Committee has clarified that the core content of the right to health includes the provision of medicines on the WHO list of essential medicines (this includes ARVs). As discussed above the cost of purchasing and providing access to ARVs is beyond the financial reach of many countries and thus to provide such treatment they must engage in international assistance and cooperation. Our research attempted to ascertain how ongoing interaction with the Global Fund impacted on the different actors’ views on the legal nature of global health obligations. It is worth recalling from our discussion in section one that legal scholars are divided as to whether such an obligation exists: some arguing yes; others yes but is not practicable; and others that it simply does not exist. We did not expect to hear high income state representatives affirm the existence of such an obligation. We found that a large majority (18) of the interviewees rejected the idea that international assistance and cooperation was based on charity. The majority did not take a firm position on the legal obligation question often blurring the line between moral obligation, legal obligation and the concept of solidarity.

One US academic noted that the structure of the Global Fund; in particular its transparency, “*has created expectations that go in the direction of obligations, not legal obligations but obligations that are more fixed without a doubt.”* A Senior European Commission official echoed this view of the Global Fund as moving beyond charity stating that “*because the funding is linked to vital services, then even less politicians are ready to be potentially accused of dropping vital treatments. So it has, I don’t know if it’s duty, but it has given them a sense of responsibility.”* Another US academic noted *“the Global Fund is more transparent, because the funds are entering into an account and there is a transparency in how those funds are used to meet the goal of funding once a country passes it over from their account to the Global Fund account.”*

These quotes suggest that both the transparent nature of the interaction and the clear link between input (i.e. funding) and results (i.e. access to treatment) have helped shaped the interpretation of the obligation. We would suggest that the Global Fund has made it easier for high income states to identify the obligation they are seeking to fulfil (e.g. X number of people on ARVs by year Y) and made their role in this process more visible. With respect to transparency the above-mentioned quote from a US academic is telling. Thus the transparency of the Global Fund decision making process helps shape the interpretation of the responsibility. Those that participate in the Global Fund open themselves up to scrutiny. The amount high income states contribute is public knowledge and NGO watchdogs like the Global Fund Observer, ensure that this information remains easily available [36]. Shining the bright light of transparency on interactions may thus impact on how they come to be interpreted in the longer term.

In terms of the link between funding and treatment (i.e. results) several interviewees echoed the European Commission official. Perhaps the Global Fund provides a partial answer to the long-standing question of how a country in a position to assist can discharge this obligation. Through ongoing interaction at the Global Fund Board decisions are taken as to what types of proposals will be funded in the field of HIV, tuberculosis and malaria. These decisions may be viewed as interpretations of the international community’s common but undifferentiated responsibility for a limited portion of global health rights. Thus the ongoing interaction has led to a new interpretation and the Global Fund can act as the vehicle to help discharge this limited obligation and countries that participate know their participation is visible in the international community.

A Belgian elected representative noted that in theory there is a legal obligation, citing a recent parliamentary resolution as further proof of the recognition of the obligation. However the individual noted the practice is different stating, *“when it comes to implementing we are not doing so well.”* This view was echoed by a Belgian civil society actor who stated, “*Well, for the moment, the contributions are still voluntary. So the whole issue on really taking responsibility is still open, I would say, except for the public opinion and so on that you can apply on it. I think for the moment it does not work at all.”*

The majority of the non-state representative respondents (e.g. academics, NGO representatives) viewed contributions to the Global Fund as a legal obligation but noted that their respective governments did not see it this way. Several statements suggest that there is something about interaction with the Global Fund that has changed the perceptions and actions of certain actors, e.g. the European politicians who may now feel more responsible for decisions relating to global health obligations We would argue that our interviews and research suggest that the process of interaction and interpretation related to the Global Fund has not (yet) led to the social or political internalization of global health obligations as legal obligations as opposed to matters of charity or political choice.

#### Shared responsibility, implying that a form of burden sharing is needed

As mentioned above the issue of how much a particular state owes to another state is a highly complex unresolved issue. The General Comment does not provide useful guidance. The Global Fund pools high income state resources which allows for a form of burden sharing. Unfortunately attempts to develop a burden sharing template were abandoned. As noted above the Global Fund is highly transparent so activists know what their governments have pledged and whether or not they have kept their promises. High income state governments can also point to their support to gain political goodwill.

We wanted to understand whether the experience of civil society working with high income states and multiple other stakeholders on the Global Fund, including the Board, would contribute to a sense of burden sharing. We also sought to understand whether or not they saw the Global Fund as an appropriate tool for discharging a common responsibility. We wondered how the process of interaction and peer-pressure on countries to pay their pledged contributions would impact on the notion of a shared responsibility. Perhaps the interactions would lead to a new interpretation of how to discharge a common but undifferentiated responsibility? Would the transparency of the Global Fund allow activists to hold high income states to pay what they pledge perhaps leading to the internalization of this responsibility?

We asked the interviewees whether their experience with the Global Fund has fostered a sense of burden sharing between high income states. A representative of an American NGO noted that “*There was a premise in the original formulation of the Global Fund that the US would pay a fair share of the global cost, and that is was based on some other equitable contribution assessments*.” A European NGO representative noted that *“One of the instruments in which the burden sharing is doing very good work is the Global Fund. It is a very concrete institution with a process that permits a kind of accountability. It’s not enough but it’s kind of a base.*” One former member of the US administration stated that *“I think the notion of peer pressure is a figment of people’s imagination*.” Another European NGO representative commented on the limitations of peer pressure “*why should France pay double of their fair share because Japan is not paying, this kind of pressure could be more normal, more accepted in diplomacy because in my conversation with some Spanish diplomats they say, “I can’t say Japan oh pay because I paid.” And I think it’s first of all, it’s an issue of culture.”* One US NGO representative identified a risk with pooling cautioning “*there’s a risk that if a country is providing what they should be providing and everyone else is providing a whole lot less, that that one country will have an excuse for scaling back*.”

The majority of interviewees made clear that burden sharing and peer pressure, both of which flow from the Global Fund model, are unique key elements of the interaction between stakeholders. One US NGO representative commented on how the pooling mechanism itself generates a sense of obligation observing, “*So my sense is that yes, I think the Global Fund mechanism makes more sense because particularly if there’s a system of obligations as opposed to a system of donations, which is very, very different*.”

The responses suggest that this process of interaction changes the interpretation and the conversation surrounding who owes what to whom in terms of contributions. Further, what came through clearly from the interviews was that the transparency of the Global Fund process allows advocates to keep up their pressure on the high income states. As no burden sharing targets have been agreed it is hard to argue that burden sharing has been internalized at any level.

#### Countries that lack domestic capacity

This issue goes to the heart of who the primary duty bearer is and how to strengthen the ability of countries that lack domestic resources to fulfil rights. As noted above, from an international health perspective the Global Fund response should be driven by countries that lack resources. In reality, countries that are unable to fulfil their obligations relating to three specific diseases can submit nationally developed plans to the Global Fund. However the plans must fulfil Global Fund criteria.

In this phase of our analysis we moved beyond assessing whether interaction had helped to generate a new interpretation of how to facilitate countries that lack domestic capacity to fulfil their obligations ^48^. Here we sought to understand how interaction at the Global Fund might have impacted on the issue of national ownership and how this concept was interpreted and even internalized. We sought to understand whether the Global Fund model was viewed as more country driven by high income state actors.

One Belgian NGO representative commented that *“The Global Fund is open, much more open to the demands of the country, the needs of the country and is less, restrictive in what can be requested…So it’s much more flexible and much more contributing really to differences on the field to the specific possibilities of getting recurrent funds financed.”* In discussing the Global Fund in-country presence one EU NGO representative noted, *“The CCM works better in some countries than in others but that is a reflection of the political realities of those actual countries in the way in which they allow and empower their citizens overall not just within the health policy making.”* A US NGO representative commented, *“The Global Fund includes so much national level participation, including civil society, it’s a way of building sustainability at the country level. And if it’s done right, increasing local capacity that ultimately ideally would decrease the need for international assistance. So I do think that the Global Fund presented donor states, including the US government, with an alternate way of viewing their engagement, so that it’s not just charitable but that it’s also ideally more empowering.”*

If one consequence of interaction is the interpretation of how to actualize human rights norms the interviews suggest that the flexibility and transparency of the Global Fund allow for debate about national responsibility including the difficult issue of disparities between states in terms of both ability and willingness to engage with their obligations. We would argue that the internalization of the norm of country ownership has progressed due to the ongoing interaction and interpretation process. Further, that norm is more internalized at the political level that is accepted by political elites than a social or legal norm. This process can not be disconnected from concurrent developments including the Paris Declaration on Aid Effectiveness which aims at increasing country ownership ^49^. Thus we would not argue that the Global Fund was the driver of change but that it played a role in the transnational legal process that has helped a new norm emerge.

#### ‘Fulfil core and other obligations’ → not limited to AIDS, TB and malaria

As noted in the discussion on the right to health above, realizing the right to health for all requires that States parties take appropriate actions relating to the right to health and the social determinants of health including sanitation and education. Thus a disease specific model, like the Global Fund, as an approach to achieving the right to health clearly has significant limitations and potentially negative implications ^50^. Interaction at the Global Fund had for some highlighted the contradictions of a disease focused approach to realizing the right to health.

The large majority of interviewees clearly recognized the limitations of a three disease model yet appeared able to look beyond this limitation. One EU NGO representative summarized what we heard from a majority of interviewees, “*When we start to talk about the people with HIV having a right to life and a right to health, we of course cannot speak only about HIV. You need to speak more broadly about the right to health there. Because why should people with HIV have a special right if people with Malaria or children dying from diarrhoea and so on. Why should HIV be special in that sense?”* An EU official noted; “*we have been consistently trying to support the progressive move of the Global Fund to go beyond these diseases*.” A US NGO representative noted*, “with the whole addition of the health system strengthening into this mix it raises that contradiction because how can you have indicators around health system strengthening that are health system oriented but with HIV money. And yet you’re expecting to show the HIV results as well and if you go through a vertical financing mechanism and a vertical reporting mechanism, how do you build up the wider health system?”*

One EU NGO representative cautioned against expanding the Global Fund model arguing that the Global Fund is far from pursuing a rights based approach “*I mean you have the classic example of the government that gets funding to look at HIV AIDS, with regards to communities of men who have sex with men, and has a policing system that criminalizes their actions, and a practice of basically violating their rights often through police brutality.”* Another EU NGO representative also expressed concern about expanding the mandate of the Global Fund noting, “*Perhaps we must be cautious therefore in thinking about a Global Fund for health because what motivates high income states with the same type of input and expected outputs from those inputs within a social Global Fund, do we want that form of quasi imperialism to rule the social sector so strongly? And we must be brutally honest that the reason the big states continue to support the IMF and continue to support the Bank is the same reason that they want to seek positions on the Security Council, it is all about the power and influence which is also why the emerging economies have wanted to have a role within the Bretton Woods institutions.”*

The majority of interviewees suggest that the stakeholders have not internalized the view that the Global Fund, as currently structured, is a tool for fulfilling the core contents relating to the right to health. However, the majority recognized its value for the three diseases and several mentioned the potential of the Global Fund approach for fulfilling the right to health more broadly. Thus in this instance repeated interaction advanced an interpretation that the Global Fund should not be limited to three diseases as this is a limited understanding of what global health obligations entail.

### The Global Fund and norm creation

The precise nature and scope of global health obligations is unclear and needs to be better understood. As such neither states in a position to cooperate and assist (high income states) nor those with a duty to request assistance and cooperation to fulfil their obligations fully understand the implications of global health obligations. However, as discussed above, ensuring universal access to anti-retroviral treatment (ARVs) for people living with HIV is clearly a core obligation related to the right to health that many states can not fulfil due to resource constraints. The Global Fund was established as a vehicle to finance such treatment. Through the process of interaction at the Global Fund Board, in negotiating, implementing and assessing grants we would argue new social and political norms were created. Social norms that recognized the obligation to provide universal access to ARVs and political norms recognizing the potential of a burden sharing approach to achieve this aim ^51 and 52^. Further, our research suggests that different stake holders have, to differing degrees; come to internalize these new norms through ongoing engagement with the Global Fund. However the internalization of these norms by high income states does not appear to have occurred.

Using a transnational legal process approach to understanding how the global legal system develops encourages us to value the impact of interaction and internalization on norm creation and ultimately compliance. Koh argues that the predictive capacity of transnational legal process pushes us to conclude that nations will come into compliance with international norms if transnational legal processes are aggressively triggered by other transnational actors forcing interaction in international forums capable of generating norms that are then internalized [16], [7], [8].

However if an enhanced degree of political responsibility is internalized this can shape both political practice and over time lead to increased commitment to the norm and thus in the long-term shape the approach to the obligation, i.e. as legal not simply moral, and lead to its legal internalization. Few high income states appear to have sufficient social and political internalization of the international cooperation norm as to internalize it legally through legislation ^53^. However the act of legally enshrining the international cooperation norm does not necessarily imply that future international cooperation will be aimed at fulfilling human rights obligations beyond borders.

We would argue that through continued interaction with the Global Fund and related constituencies some high income states have started to understand how they can discharge one element of their global health obligations. However the global economic downturn that began in 2008 has challenged the transnational legal process allowing states to step back from political internalization. It is here that advocacy and in some cases national or transnational public law litigation have a key role to play in harnessing the political will required to shape government policy and ultimately push those governments in a position to assist towards legal internalization and compliance.

## Conclusions

In this paper we have argued that the tendency of legal scholars to focus solely on compliance related behaviour and speech, fails to capture the normative effect of international human rights law on the current practice of global health actors, as it moves along the path towards or away from acceptance of global health obligations.

As noted above, the following statement by Paul Hunt framed our interviews “if there is no legal obligation underpinning the human rights responsibility of international assistance and cooperation, inescapably all international assistance and cooperation is based fundamentally upon charity. While such a position might have been tenable 100 years ago, it is unacceptable in the twenty-first century." [10]. The Committee has asserted that international cooperation for development is a binding obligation under the ICESCR [4]. This authoritative interpretation has been largely rejected by many high income states that fear its potential implications ^54^. Hunt et al. note that many low income states agree with the Committee’s interpretation that the human rights responsibility of international assistance and cooperation places binding legal obligations on states; those requiring assistance and those in a position to assist [19].

We suggest that our case study shows how the transnational legal process of interaction, interpretation and internalization of international human rights norms can lead to enhanced compliance in the absence of new “hard law”. In particular our research has suggested that outright resistance to the international legal obligation to cooperate and provide development assistance to realize the right to health, may be eroded if states in a position to assist can be shown how this obligation can be discharged. Through ongoing interaction at the Global Fund (multiple meetings over the past decade) high income states came to understand how this limited element of their obligation could be discharged. As such they came to a new interpretation of how to engage with their obligation, namely contributing to the Global Fund. The engagement of civil society in this process was important as it acted as a watchdog for both high income states and low income states. The albeit limited transparency of the Global Fund allowed for this type of interaction with the evolving norm. Thus our research supports Berman’s contention that, “to the extent that international human rights are now an important element of global legal consciousness; it is because of a long process of rhetorical persuasion, treaty codification, and other forms of “soft law” slowly changing the international consensus, not because of positivist decree.” [11]. No new hard law has been proclaimed but a consistent pattern of interaction and pledging new funds suggests some high income states are moving towards interpreting their funding of the Global Fund as a vehicle for discharging their responsibility related to three global diseases.

We have argued that a process focused lens suggests avenues for exploring how acceptance and respect for norms evolves. Thus even while uncertainty as to the precise scope of global health obligations exists we suggest it is worth examining state practice and interaction and that the knowledge gained can help shape research and advocacy agendas ^55^. As such, we hope that our research provides a limited response to Schrecker et al’s plea for more research on how the acts, policies and omissions of rich, powerful countries affect economic and social rights beyond their borders ^56^.

Although we have focused our attention on how complex international human rights obligations can be operationalised we do not intend to suggest that state and non-state actors have or have not become more compliant with their international human rights obligations. International human rights law has a key role to play in addressing these violations by identifying the entitlements of individual rights holders and the corresponding obligations of diverse duty bearers. The strength of human rights lies in its empowerment of people to claim their rights and to hold duty bearers accountable for failures but this requires the political will to develop robust accountability mechanisms [37].

Drawing from the logic underpinning Cover’s defence of the Nuremberg trials which was based on “the capacity of the event to project a new legal meaning into the future” [35] we question whether the Global Fund has created a degree of “new legal meaning” in the area of global health obligations. The future direction of the Global Fund is uncertain but as Bob Deacon wrote: “The Global Fund might be taken as an example of how global innovative redistribution mechanisms are being established” [38].

Although far from perfect, the Global Fund has highlighted the power of transparency and multi-stakeholder partnerships in creating new legal meaning including how to engage in global health cooperation. This legal meaning is free for others to build on in the future.

## Endnotes

^1^ In 2000, the international community adopted eight Millennium Development Goals (MDG) with targets to be reached by 2015. MDG 5 calls on the global community to reduce maternal mortality by three quarters by 2015 and to achieve universal access to reproductive health. The Human Rights Council identifies a range of human rights directly implicated by maternal mortality and morbidity, namely, the “rights to life, to be equal in dignity, to education, to be free to seek receive and impart information, to enjoy the benefits of scientific progress, to freedom from discrimination, and to enjoy the highest attainable standard of physical and mental health, including sexual and reproductive health”. Resolution 11/8 (para. 2), United Nations Human Rights Council [http://www2.ohchr.org/english/bodies/hrcouncil/docs/14session/A.HRC.14.39_AEV-2.pdf]

^2^ As was evident in negotiations surrounding the Optional Protocol to the ICESCR detailed in [39].

^3^ For a detailed examination of extraterritorial obligations please see ref. [40]. For an examination of the issue of international assistance and cooperation and health and human rights obligations beyond borders please see the volume of ref. [41].

^4^ Please see the reports of Paul Hunt and Anand Grover [http://www.ohchr.org/EN/Issues/Health/Pages/AnnualReports.aspx]. Hunt’s reports included missions to pharmaceutical companies as well as a focus on more neglected right to health issues like mental disability and maternal mortality. Grover has also chosen to explore his mandate broadly examining guidelines for pharmaceutical companies in relation to access to medicines. [http://www.ohchr.org/EN/Issues/Health/Pages/SRRightHealthIndex.aspx].

^5^ We shall use the term global health obligations to refer to the international legal obligations found in international human rights treaties including the International Covenant on Economic, Social and Cultural Rights (ICESCR). The scope of these obligations is not yet fully defined but has been examined by the UN Special Rapporteur on the Right to Health, the Committee on Economic, Social and Cultural Rights and other legal scholars. The recently launched Joint Learning Initiative on National and Global Responsibility for Health (JALI) was formed to rigorously and systematically address such issues including clarifying more precisely the responsibility of all states and that of the international community with respect to the right to health and “clarify the essential package of health goods and services to which all human beings are entitled as part of their right to health.” [42].

^6^ For some scholars compliance is the key measure for evaluating any theory of international law. Many wealthy powerful states and non-state actors violate human rights beyond their borders on a daily basis. In no way do we intend to suggest that our results show there is increasing respect for the rights of people in third states or compliance with international human rights law [43].

^7^ As noted above General Comment 14, the work of the Special Rapporteurs on the right to health and the research undertaken by the JALI offer guidance on this issue , see ref. [4] and (4,5). Further authoritative guidance regarding states’ extraterritorial obligations can be found in the Maastricht Principles on Extraterritorial Obligations of States in the area of Economic, Social and Cultural Rights which were agreed in October 2011 by a group of prominent international legal scholars. [http://www.icj.org/dwn/database/Maastricht%20ETO%20Principles%20-%20FINAL.pdf]

^8^ See article 38.1 of the Statue of the International Court of Justice which states that the primary sources of international law are international treaties and conventions, customary practices of states accepted as law and general principles of law common to most legal systems. Statue of the International Court of Justice**.** 59 Stat. 1055, 33 U. N.T.S. 993; 1945.

^9^ In January 2011 a newspaper article detailing small scale grantee/implementer corruption led some high income states to delay paying pledged funds to the Global Fund. In response the Global Fund created an Independent Inspection Panel which undertook a wide-ranging review of Global Fund’s operations. The wide-ranging recommendations identified many areas for improvement but have been criticized for their breadth. Most high income states have responded positively to the report. Far more important was the decision by the Global Fund Board to “cancel” (postpone) a pledging round (Round 11). The ongoing global financial crisis was blamed for this decision which will have a huge impact on scaling up treatment for HIV and thus result in the premature death of many. At this stage it is not possible to determine whether or not this decision reflects a lack of confidence in the Global Fund model or is truly a result of the crisis. For details see Aidspan: *The Global Fund Observer*, Issue 158, 20 September, 2011. [http://www.aidspan.org/documents/gfo/GFO-Issue-158.htm]

^10^ For methodological background see ref. [44]. For an example of this approach see ref. [45].

^11^ Belgium has ratified the ICESCR, the United States has signed but not ratified the ICESCR. All European Union member states have ratified or acceded to the ICESCR but the European Commission is not a party to the ICESCR. For details concerning contributions and pledges to the Global Fund please see:[http://www.theglobalfund.org/en/about/donors/public/]

^12^ Whether or not the internalization of a norm should be viewed as compliance like behaviour or jurisgenerative, i.e. creating a new norm that is to be complied with is beyond the scope of this essay.

^13^ The UN Special Rapporteur on the right to food, Olivier De Schutter, observes that the International Court of Justice encourages the recognition of the Universal Declaration of Human Rights as a general principle of international law noting that as far back as *the Corfu Channel Case (United Kingdom of Great Britain and Northern Ireland-Albania)(I.C.J. Reports 1949, 4 at 22*) the courts mentioned “obligations… based … on certain general and well-recognised principles” cited in ref. [46]. Also see ref. [47].

^14^ World Health Organization: *Constitution of the World Health Organization*, 14 U.N.T.S. 186, 1946. The preamble proclaims that the enjoyment of the highest attainable standard of health is a fundamental right of every human being without distinction, and that governments are responsible “for the health of their peoples which can be fulfilled only by the provision of adequate health and social measures.”

^15^*Universal Declaration of Human Rights*, G.A. Res. 217 (III) 1948, UN Doc A/810, p. 71. Article 25.1 states that, “everyone has the right to a standard of living adequate for the health and well-being of himself and of his family, including food, clothing, housing and medical care and necessary social services.”

^16^*International convention on the elimination of all forms of racial discrimination,* G.A. Res. 20/2106; UN GAOR 2106 (X) 1966: U.N.T.S. 195; *International covenant on economic, social and cultural rights*, G.A. Res. 21/2200A, UN GAOR Supp. (No. 16) at 49. UN Doc A/6316 1966. *Convention on the elimination of all forms of discrimination against women,* G.A. Res. 34/180, UN GAOR, 34^th^ Sess., Supp. No. 46 at 193 UN Doc.A/34/46 1979. *Convention on the rights of the child*, G.A. Res. 44/25, UN GAOR, 44^th^ Sess., Supp. No. 49, at 166, UN Doc. A/44/25 1989. *Convention on the rights of persons with disabilities*, G.A. Res. 61/106; 2006, UN GAOR, 61^st^ Sess. Annex I, UN Doc. A/RES/61/106 (2006).

^17^*Charter of the United Nations*, signed 1945, 59 Stat. 1031, *entered into force* Oct. 24, 1945. [http://www.un.org/en/documents/charter/chapter9.shtml] Article 55 (c) notes that the UN shall promote “universal respect for, and observance of, human rights and fundamental freedoms for all without distinction as to race, sex, language or religion.” Article 56 also uses mandatory language to refer explicitly to the duties of members to take joint and separate action to achieve the purposes set out in article 55.

^18^ Article 2.1 of the ICESCR states “Each State Party to the present Covenant undertakes to take steps, individually and through international assistance and co-operation, especially economic and technical, to the maximum of its available resources.”

^19^ Articles 4 and 23- specifically referencing the importance of international cooperation and assistance in, *inter alia,* achieving the right to health. Article 23(4) of the *Convention on the Rights of the Child (CRC)*, affirms that “States Parties undertake to promote and encourage international cooperation with a view to achieving progressively the full realization of the right recognized in the present article. In this regard, particular account shall be taken of the needs of developing countries”. For a rich analysis please see ref. [48].

^20^ Article 32 of the *Convention on the Rights of Persons with Disabilities* notes that States Parties, “recognize the importance of international cooperation and its promotion, in support of national efforts for the realisation of the purposes and objectives of the present Convention, and will undertake appropriate and effective measures in this regard.”

^21^ Commenting on this issue in 1987 (prior to the 1990 release of General Comment 3 on the nature of States parties’ obligations), Philip Alston noted, “A logical implication of the use of the terminology of rights. In other words, there would be no justification for elevating a claim to the status of a right (with all the connotations that concept is generally assumed to have) if its normative content could be so indeterminate as to allow for the possibility that the rights holders possess non minimum entitlement, in the absence of which a State Party is to be considered in violation of its obligations.” p. 352–353 in [49].

^22^ For example Article 24(4) of the CRC recognizes that as with other economic and social rights, the rights enshrined in the CRC will be achieved progressively and not immediately.

^23^ The minimum core approach as developed by the Committee has generated controversy. Katharine Young provides a challenging critique of the minimum core approach see ref. [50]. John Tobin labels the minimum core elucidated in General Comment 14 to be “unprincipled and impractical. The long list of measures required of states is so onerous that few states, if any, are likely to adopt such an approach.” (p 48) [51].

^24^ An example of the importance of the core obligation to develop a national health plan is evidenced in the work of major global health actors; for example, the Health Systems Funding Platform (bringing together the GAVI Alliance, the Global Fund and the World Bank and coordinated by the WHO) requires that a national health plan (assessed and refined through a joint assessment process) serve as the basis for all health related funding that it coordinates. [http://www.gavialliance.org/resources/HSF_Platform_FAQ_15.01.2010.pdf]

^25^ Specifically paragraph 47 affirms “a State party cannot, under any circumstances whatsoever, justify its non-compliance with the core obligations … which are non-derogable”

^26^ For a rich analysis of potential perils associated with the minimum core approach please see ref. [52]. They argue that “while we see certain problems with the minimum core approach, and in particular with the idea of non-derogability, we believe that a cautious continuation of the doctrine is warranted at the present time. However, it must be understood in context.” p. 495.

^27^ The 40 USD estimate can be found in ref. [53]. The contents and cost of a basic package are disputed but even a low estimate is far above what the world’s poorer countries can afford. For a recent examination of the costing issue please see ref. [54].

^28^ “Such minimum core obligations apply irrespective of the availability of resources of the country concerned or any other factors and difficulties.” Para. 9, ref. [55]. These Guidelines were prepared by a group of legal experts to outline the emerging consensus within the legal community in the late 1990s as to the specifics of state responsibility and accountability under the ICESCR.

^29^ Margot Salomon has advanced a more expansive understanding of the obligation of international cooperation requiring, inter alia, wealthy states to address the structural causes of world poverty, see in particular pages 99–104 [22].

^30^ This obligation to provide international assistance is not a bottomless pit. The financial cost of this obligation has been tied to the high income state’s available resources and its obligations to its citizens. Striking the right balance is a complex politically sensitive issue that requires negotiation. The 0.7 percent of GDP goal is one target used by the Committee when examining the conduct of high income state countries. However it is not simply the volume of assistance that is important but also the way in which it is provided; i.e. is it used in a manner which supports or undermines human rights in a low-income state?

^31^ The pitfalls of assessing when a state has expanded the maximum of available resources have been explored by numerous commentators including ref. [56]. Further, establishing the appropriate benchmarks and indicators to assess in determining whether a state is unwilling or unable is contentious. As Tomasevski has argued in some cases it may be more about policy than poverty. See ref. [57]. Ensuring that the human rights of residents of an unwilling state are fulfilled requires creative solutions from the international community such as cooperating with legitimate civil society groups and NGOs. They also suggest the need for a deeper analysis and reform of structural impediments to the realisation of rights as suggested by Salomon, see ref. [22].

^32^ Unlike “older” causes of premature death including; malaria, tuberculosis, diarrhoea and maternal mortality, when the first cases of AIDS appeared they posed a new challenge to the World-how to diagnose this new disease, the cost of investing in research to find a treatment and cure etc. Once life-saving treatment was available another challenge arose. Would treatment be made available to those in resource poor settings-the countries most affected by HIV/AIDS? Citing the United States Centers for Disease Control Morbidity and Mortality Weekly Report (5 June, 1981) [25].

^33^ Jonathan Mann’s ground-breaking research on AIDS led him to advocate and research on the linkages between health and respect for human rights. As Head of the World Health Organization’s Global AIDS Program his advocacy and research on the connection between respect for human rights and vulnerability to HIV greatly influenced the response to HIV/AIDS. See ref. [58]. Following his death the muli-layered implications of the connection between health and human rights have been rigoursly explored by scholars including notably Sofia Gruskin. See ref. [59,60]

^34^ For further details see the Global Fund’s website [http://www.theglobalfund.org/en/whoweare/?lang=en.]

^35^ As noted above the Global Fund has no country-level presence and is designed to operate through the CCM, a multi-sectoral country level partnership.

^36^ Given the recent funding crisis at the Global Fund it can be argued that this inclusive structure is proving a step to far for some high income states that prefer being in the driver’s seat.

^37^ For an example please see ref. [61].

^38^ As Benedict Kingsbury argues convincingly compliance is not simply “‘correspondence of behaviour with legal rules” [62]. He posits that “Concepts of ‘compliance’ depend upon understandings of the relations of law, behaviour; objectives, and justice.” (at 346) Thus compliance is not a free-standing concept but derives its meaning from different theories each of which impacts on the definition of compliance.

^39^ Theories of international legal compliance remain underdeveloped as traditionally such issues were the purview of international relations scholars, not international lawyers.

^40^ See the works of international relations scholars like [63] and the work of international lawyers like Kenneth Abbott, e.g., [64].

^41^ The work of Anne-Marie Slaughter stresses the importance of a state’s domestic structure and the strength of the rule of law as determinative factors in compliance with international law, see e.g. [65].

^42^ Koh’s theory evolved from his practical experience in ‘transnational public law litigation’ against both US and other foreign officials on behalf of victims of human rights abuses see e.g. [66].

^43^ For a description and analysis of the forces shaping this process see ref. [67]. Koh’s reference to the US should not be understood as limiting this analysis to the US but rather as exemplary for domestication in domestic law and policies.

^44^ The New Haven School of International Law contributed greatly to ground breaking scholarship on legal pluralism arguing that through the interaction, interpretation and enforcement behaviour of multiple diverse communities transnational law becomes important and even transformative [68]. See also the very influential work of the Chayeses arguing that compliance with international law is often best achieved through a managerial approach rather than through sanction [69].

^45^ Goodman and Jinks’s work on the role of the acculturation process in influencing states compliance with human rights law takes transnational legal process one step further see ref. [70]. In this article they define acculturation as “the general process by which actors adopt the beliefs and behavioral patterns of the surrounding culture” (at p 726). Like Koh their work responds to a trend in international law and human rights scholarship to focus on coercion and persuasion based theories of international law moving beyond asking why nations comply with human rights law to asking how we might better design regimes so that they enhance and facilitate respect for human rights.

^46^ Koh cites the complex history of the UK’s incorporation of the European Convention on Human Rights into domestic law as such an example.

^47^ For a discussion of the ‘jurisgenerative process’ see ref. [71]. Arguing for example that “the *position* that only the state creates law… confuses the status of interpretation with the status of political domination.” (p. 43)

^48^ One area that we identified as problematic was the fact that the Global Fund’s fourth largest recipient in terms of cumulative disbursement of grants is China. [http://www.globalhealthfacts.org/data/topic/map.aspx?ind=60]. It is difficult to argue that China is as a country that lacks domestic capacity to fulfil its obligations. We thus chose to focus on the potential of the Country Coordinating Mechanism as an example of a new interpretation or approach to discharging this element of the core content.

^49^ The Paris Declaration on Aid Effectiveness, 2005 and the Accra Agenda for Action 2008 aim at changing county behaviour and increasing the effectiveness of aid to help achieve the MDGs. [http://www.oecd.org/dataoecd/30/63/43911948.pdf]

^50^ The impact of GHIs on the health systems of low income countries is a much debated topic and the evidence is mixed. In some countries disease specific interventions may have weakened other health services whereas in other countries disease specific interventions appear to have strengthened the wider health systems [72].

^51^ The reaction of civil society and activists to the “cancellation” of a recent pledging round for the Global Fund suggests that low income country activists and high income country activists now view cooperation with the Global Fund as a norm and are pushing low-income governments to step up their health assistance and for high-income governments to fund the Global Fund. See [http://www.aidspan.org/documents/gfo/GFO-Issue-174.htm] [http://www.aidspan.org/documents/gfo/GFO-Issue-170.pdf]

^52^ Politicians in many high income state countries choose to use the Global Fund as the main vehicle for funding ARV treatment in the Global South instead of shifting towards bilateral aid structures to provide such funding.

^53^ In its 2010 election manifesto the British Conservative party pledged to enshrine the 0.7% of GNI to ODA in law but as of March 2012 the Conservative led British Government had failed to do so.

^54^ The more conservative approach discussed by Skogly see ref. [14], namely; the obligation to ensure that their development assistance does not violate economic and social rights, is more in line with current high income state policy e.g. of the UK’s DFID and Sweden’s SIDA.

^55^ Balakrishnan Rajagopal calls for international lawyers to engage in social movements and activism and for them to use international law and arenas to expand the space available for transformative politics and move away from its Western, elitist, male-centered imperial past [73].

^56^ In discussing the potential of human rights Schrecker et al. assert, “their theoretical strength as a challenge to the norms of the global marketplace” and stress “the importance of collaboration between those working in human rights and in social determinants of health to define common objectives and develop research programs and advocacy strategies for moving from compelling theory to effective practice” [74].

## Competing interests

The authors declare they have no competing interests.

## Authors’ contributions

RH, GO and WV conceived the study. RH and GO conducted the interviews. RH and GO analysed the data. RH drafted the manuscript and GO and WV revised the manuscript. All authors read and approved the final manuscript.

## Authors’ information

Rachel Hammonds (JD) is a researcher in the Public Health Department of the Institute of Tropical Medicine in Antwerp, Belgium and a member of the research group Law and Development at the Faculty of Law, University of Antwerp, Belgium

Gorik Ooms (PhD) is a researcher in the Public Health Department of the Institute of Tropical Medicine in Antwerp, Belgium and an adjunct professor of law at Georgetown University, Washington, DC. He is the former executive director of Médecins Sans Frontières Belgium.

Wouter Vandenhole (PhD) teaches human rights law and holds the UNICEF Chair in Children’s Rights at the University of Antwerp, Belgium. He is a member of the research groups Law and Development and Government and Law at the Faculty of Law, University of Antwerp, Belgium.

## Pre-publication history

The pre-publication history for this paper can be accessed here:

http://www.biomedcentral.com/1472-698X/12/31/prepub
